# Personal

**Published:** 1899-01

**Authors:** 


### PERSONAL.

DR. WESLEY C. EARL, of Buffalo, announces that he has removed his office and residence from 1541 Niagara street to 147 Lafayette avenue, corner of Herkimer street. Hours: 9 to 10 a. m. 1 to 3 and 7 to 8 p. m. Telephone, Bryant 2366.

---

DR. DAVID WEBSTER, New York, spent a day or two in Buffalo, about the middle of December, as the guest of Dr. R. H. Satterlee, of Delaware avenue.

---

DR. JOHN N. GOLTRA, of Buffalo, acting-assistant surgeon, U. S. Army, was directed, under date of December 1, 1898, to proceed to this city for annulment of his contract.

---

DR. BINA POTTER VANDENBERGH, of Buffalo, announces to her friends and patients that she is permanently settled in this city with offices at her residence, 122 North Pearl street.

---

DR. CHARLES P. KNOWLES, of Buffalo, announces to the profession that he has removed from 878 to 915 Elmwood avenue. Hours: 8 to 9.30 a.m.; 1 to 3 p. m. and 7 to 8 p. m. Telephone, Bryant 2001.

